# Ontology-based identification and prioritization of candidate drugs for epilepsy from literature

**DOI:** 10.1186/s13326-021-00258-w

**Published:** 2022-01-24

**Authors:** Bernd Müller, Leyla Jael Castro, Dietrich Rebholz-Schuhmann

**Affiliations:** 1 ZB MED - Information Centre for Life Sciences, Gleueler Str. 60, Cologne, 50931 Germany; 2 University of Cologne, Albertus-Magnus-Platz, Cologne, 50923 Germany

**Keywords:** Epilepsy, Ontology, Open discovery process, Knowledge discovery, Top-k, Enrichment analysis, Drug repurposing, Drug discovery, Information extraction, Text mining

## Abstract

**Background:**

Drug repurposing can improve the return of investment as it finds new uses for existing drugs. Literature-based analyses exploit factual knowledge on drugs and diseases, e.g. from databases, and combine it with information from scholarly publications. Here we report the use of the Open Discovery Process on scientific literature to identify non-explicit ties between a disease, namely epilepsy, and known drugs, making full use of available epilepsy-specific ontologies.

**Results:**

We identified characteristics of epilepsy-specific ontologies to create subsets of documents from the literature; from these subsets we generated ranked lists of co-occurring neurological drug names with varying specificity. From these ranked lists, we observed a high intersection regarding reference lists of pharmaceutical compounds recommended for the treatment of epilepsy. Furthermore, we performed a drug set enrichment analysis, i.e. a novel scoring function using an adaptive tuning parameter and comparing top-k ranked lists taking into account the varying length and the current position in the list. We also provide an overview of the pharmaceutical space in the context of epilepsy, including a final combined ranked list of more than 70 drug names.

**Conclusions:**

Biomedical ontologies are a rich resource that can be combined with text mining for the identification of drug names for drug repurposing in the domain of epilepsy. The ranking of the drug names related to epilepsy provides benefits to patients and to researchers as it enables a quick evaluation of statistical evidence hidden in the scientific literature, useful to validate approaches in the drug discovery process.

## Background

Drug repurposing provides an alternative approach to drug discovery by identifying novel disease indications for already approved pharmaceutical compounds, reducing time and risks involved in the regular process of drug discovery [1]. By the year 2020, approximately 30 *%* of the US Food and Drug Administration (FDA) approved drugs and vaccines have been repurposed wrt their original disease indication [2]. Drug repurposing uses known information about drugs and diseases as well as complementary data sources to determine similarities across drugs and diseases and thus identify new uses for existing drugs.

### Data resources for drug repurposing

The hypothesis behind drug repurposing is that similar properties of drugs and diseases allow the inference for new application domains. The vast amount of publicly available biomedical databases provide a rich resource for factual knowledge on drug and disease-related properties that can be later used to calculate similarities.

Diseases can show similarities regarding, for instance, clinical symptoms, diagnosis, disease progression, and co-morbidities that can be used to make statements about the application of different drugs for similar diseases. There is a variety of biomedical sources providing relevant information to find such similarities, e.g. phenotypes, gene expression and gene-disease association. The database Online Mendelian Inheritance in Man (OMIM) [3] is a comprehensive resource for genetic phenotypes. Gene expression profiles are available in databases such as ArrayExpress [4] and GEO [5]. DisGeNET [6] is a database about gene-disease associations extracted from the literature and linked to database records. Ontologies provide background information that can help glue together data from different sources including semi or unstructured data such as that one coming from literature.. For example, domain-specific ontologies contain semantic relations of diseases and drugs that are not directly available in literature or databases.

**Fig. 1:**
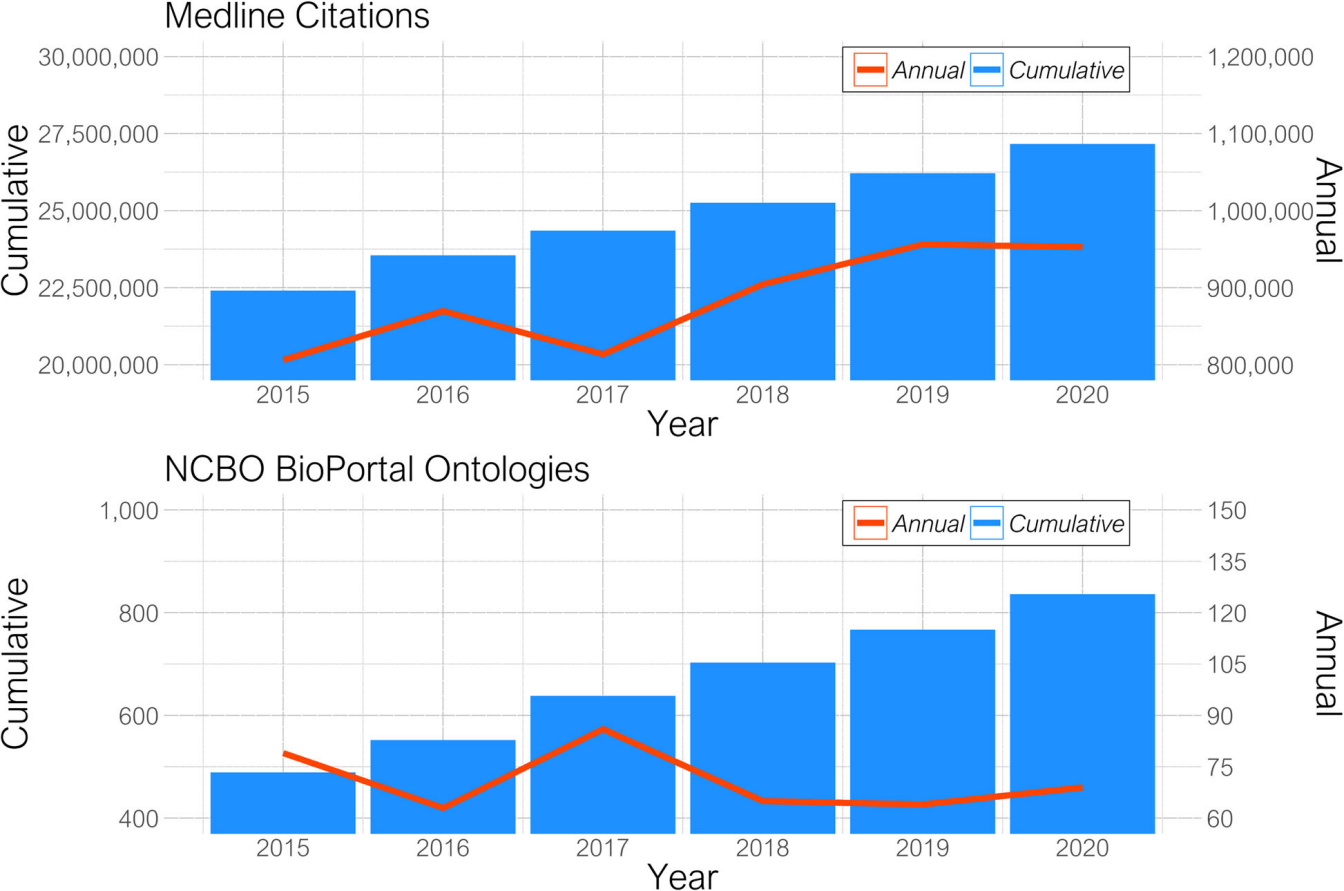
Annual and cumulative growth of citations in NLM’s Medline and ontologies in NCBO’s BioPortal from 2015 until 2020

Biomedical ontologies provide information useful to calculate semantic similarities between heterogeneous data sources beyond implicit relationships, and to connect databases with bibliographic information. Although manual curation of relevant information from literature is a practice followed by some domain specific databases, using such approach over the whole literature is not a scalable option as the published literature exceeds manual curation capacity. For instance, Medline introduced almost 1.1 million new publications in 2020 reaching more than 27 Million publications in total [7]. In parallel, the National Center for Biomedical Ontology (NCBO) BioPortal [8] adds about 75 new ontologies each year with a total of a bit more than 900 ontologies in 2020. Both annual and cumulative growth rates of Medline citations and ontologies in BioPortal are shown in Fig. 1. It is hardly possible for any researcher to keep track of their domain knowledge represented as ontologies without incorporating automated methodologies for the retrieval and discovery of relevant information from literature. The automated extraction of the drug-disease association from literature resources is a yet neglected but very valuable contribution to current drug repurposing approaches because many relationships between drugs and diseases are still buried in the free-text of biomedical publications, unavailable in biomedical databases but decipherable with the help of text mining and domain ontologies.

### Related work

Recent approaches incorporate different types of semantic information useful for the computation of association and similarity between drugs and diseases. Zhang et al. [9] used a matrix factorization method to represent drug-disease associations for the projection in low-rank spaces for the prediction of novel associations. Jiang et al. [10] developed a sparse auto-encoder as a symmetrical neural network for the prediction of novel drug-disease associations incorporating existing data sets; they include an evaluation of their approach on obesity, and lung and stomach neoplasms. Zhu et al. [11] constructed a knowledge graph for the integration of various drug knowledge bases in order to derive novel disease associations comprising a case study for the association of the anti-diabetic drug metformin to various cancer types. Despite the current efforts, the application, the application of text mining methods to compute semantic similarities has still more to offer wrt the discovery of novel relationships between drugs and diseases.

### Using term sources for knowledge discovery

Using domain ontologies for text mining on neurodegenerative diseases literature leads to successful outcomes that could be used for drug repurposing as shown on the following examples. The Alzheimer Disease Ontology has been developed as a resource for preclinical, clinical, etiological, and molecular/cellular mechanisms with a successful application in text mining for the automated extraction of comorbidities [12]. The Multiple Sclerosis Ontology has successfully been employed for the automated extraction of drug-targets with their functional biological pathways from free-text in PubMed articles and electronic medical records [13]. The Parkinson Disease Ontology has been developed for the use of text mining by automatically annotating the free-text of data sets describing gene expression profiles [14]. For other neurodegenerative diseases, including epilepsy, there are some other domain-specific ontologies that can be used for text mining and potentially for drug repurposing.

Literature-based discovery is the process of connecting "islands of knowledge" from articles published in different journals and scientific disciplines by linking the co-occurring concepts, even if they do not appear within the same document [15]. In the mid-1980s, Don R. Swanson developed the foundations of literature-based discovery for drug repurposing with the so-called ABC model [16] with discoveries of fish oil for treating Raynaud’s disease [17], magnesium for preventing migraine [18], arginine increasing blood levels of somatomedin C [19], magnesium deficiency playing a role in neurological diseases [20], the potential protective effect of indomethacin in Alzheimer’s disease [21], estrogen replacement therapy lowering the risk of Alzheimer’s disease [22], and calcium-independent phospholipase A2 playing a role in Schizophrenia [23]. The linkage of different types of concepts is conducted by using the *B-Terms* which co-occur with a concept *A* and a concept *C* while concept *A* and concept *C* do not necessarily co-occur, forming an implicit link between *A* and *C* through the *B-Terms*. The Open Discovery Process [24] is an extension of the ABC-model connecting a set of concepts *A* with a set of concepts *C* through a set of *B-Terms*.

### Using epilepsy ontologies

For the domain of epilepsy, several domain-specific ontologies have been constructed for various applications. The Epilepsy and Seizure Ontology (EpSO) [25] has been developed to extract epilepsy-related concepts from the free-text of electronic medical records and therefore categorize information on epilepsy and seizures [25, 26]. The Epilepsy Syndrome Seizure Ontology (ESSO) has been developed to capture the various historical classification systems of epilepsy [27, 28]. The Epilepsy Ontology (EPILONT) has been constructed for the translation of epilepsy and seizure information from English into Portuguese [29, 30]. The ontology Epilepsy Semiology (EPISEM) contains signs and symptoms for epilepsy syndromes and seizure types [31]. The ontology Functional Epilepsy Nomenclature for Ion Channels (FENICS) is designed to capture electrophysiological experiments on Ion Channels in the context of epilepsy [32]. To the best of our knowledge, none of these ontologies has been yet used for the automated extraction of drug-disease associations for drug repurposing.

**Fig. 2:**
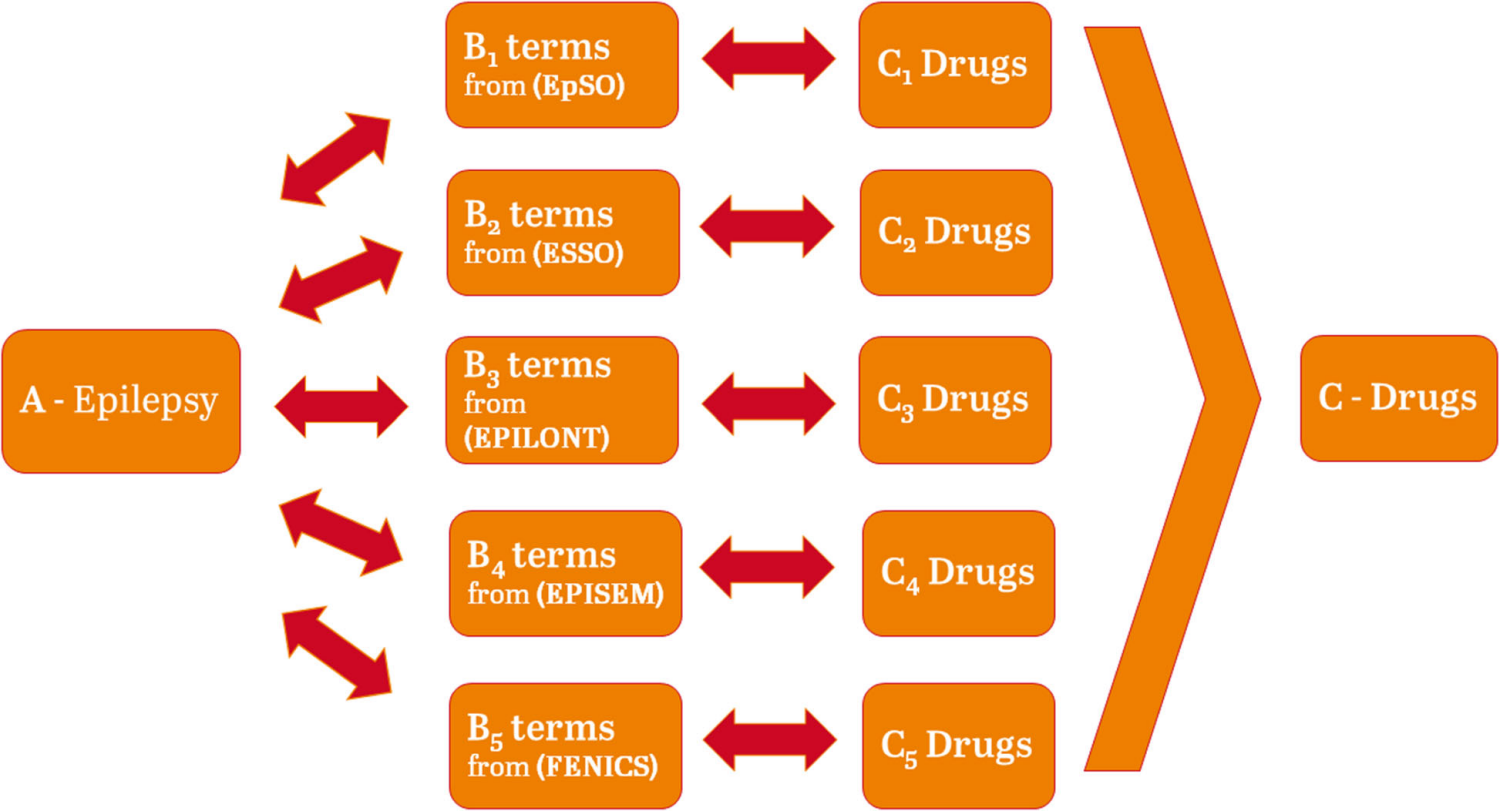
The Open Discovery Process according to Swanson’s ABC-model implicitly connecting Epilepsy (*A*) with Drug Names (*C*) through co-occurring terms from the epilepsy-specific domain ontologies (*B*−*Terms*)

In this study, we use the Open Discovery Process to connect epilepsy to drug names with the help of co-occurring *B-Terms* from epilepsy-specific domain ontologies as shown in Fig. 2. The different ontologies are considered as set of terms regarding the Swanson and Smalheiser discovery approach, going a step ahead by taking the ontologies as a whole and thus creating a large set of terms to characterize the retrieval of drug names, i.e. *C-Terms*. All available concepts from each of the considered ontologies are taken as *B-Terms*. The results of this approach could pinpoint towards novel applications of drugs such as Ketamine [33], a drug well-known for its use as anesthetic since the 60’s but nowadays also used to treat refractory depression [34] and showing efficacy for the management of refractory epilepsy [35].

The fundamental hypothesis behind this approach is that underlying malfunctioning processes are potentially shared between different neurological and neurodegenerative diseases and disorders. Therefore, drugs which are approved for one neuropathological indication could show efficacy for a different neurological indication area because the drugs modulate similar or even shared malfunctioning neurological processes. Drug repurposing for neurological drugs is especially complex for the neurological domain as "the structural complexity of the nervous system and influence of the blood-brain barrier permeability often becomes more difficult to develop new drugs in neuropathological conditions than diseases in other organs" [36]. Due to this complexity, the result sets of extracted drug names undergo a special filtering step for the selection of neurological drug names only. In any case, a combination of multiple solutions have to be brought together to achieve drug repurposing; our suggested solution helps identify candidates and it lies in the hands of the researcher performing the search / retrieval, what candidates could be taken into account for further consideration.

### Limitations

The Open Discovery Process groups drugs related to epilepsy by identifying drug names co-occurring with terms from different epilepsy ontologies in the 2021 BioASQ corpus, and thus enables the extraction of drug names in context of epilepsy even if the term epilepsy does not occur in the same document with the drug name. However, this approach fails to detect the type of the relation between epilepsy and the respective drugs. Such relation should be determined by other means which are out of the scope of the present manuscript.

### Evaluation with reference lists

In order to evaluate the extracted drug names, they are ranked according to their co-occurrence frequency and compared to several reference sets of drugs recommended for the treatment of epilepsy. For instance, Perucca and Tomson suggest drugs for the first-line treatment of epilepsy in adults [37] (reference data set known as Lancet) while Trinka and colleagues suggest various drugs for treating early, established, and refractory seizures as well as for other stages of epilepsy [38] (reference data set called DRUGSE). The Epilepsy Foundation also provides a list of medications for seizures on their website [39] (reference data set called EFO). Similarly, an updated list of drugs for the initial treatment of epilepsy in adults is available at the U2D data set [40]. As a minimum, the ranking of extracted drug names should recover drugs that are already approved as anti-epileptics or drugs that are frequently used as first-line treatment for epilepsy in the reference lists. As part of the discovery process, other types of relations between drugs and epilepsy should be found, including, for instance, drugs that could be used for treating epilepsy, drugs having some type of relation to epilepsy or drugs causing seizures as side effects.

## Results

The terms from the source ontologies are used as sets of terms to identify *B-Terms* in the documents as part of the discovery process.

### Identification of B- and C-Terms

After creating the dictionaries corresponding to drug names and terms from the epilepsy ontologies, named entity recognition (NER) was applied to the 2021 BioASQ corpus [41, 42], which contains 15,501,443 citations from Medline. Each identified term in the text either by a matching synonym or label forms a so called stand-off annotation which comprises the character offset in the document, the matched text, the length of the matched text, the label, and the source dictionary. The resulting stand-off annotations together with the aggregation of documents containing drug names co-occurring with terms from the epilepsy ontologies are stored in a MongoDB, Table 1 shows data related to the number of concepts as well as documents where *B-* and *C-Terms*, i.e. drug names, (co-)occur. The number of terms in the different source ontologies varies from a small number, e.g. 530 terms in the ontology EPILONT, up to 7,284 terms in the ontology ESSO. Numbers shown in the table indicate already that the term sets from the different ontologies have different characteristics, when serving as *B-Terms* in the discovery process.

**Table 1:** Number of concepts and synonyms for the dictionaries created from the source ontologies corresponding to drug names. Additionally, number of documents from the BioASQ 2020 corpus of 15,501,443 citations where (*B-Terms*) co-occur with (*C-Terms*)

	EpSO	ESSO	EPILONT	EPISEM	FENICS
Concepts	1,357	2,694	137	1,591	141
Unshared Concepts	87.693%	77.803%	56.204%	76.996%	100%
Shared Concepts	12.307%	22.197%	43.796%	23.004%	0%
Synonyms	3,059	7,284	530	4,847	708
Unshared Synonyms	91.01%	85.214%	81.698%	81.886%	100%
Shared Synonyms	8.99%	14.786%	18.302%	18.114%	0%
Synonyms per Concept	2.268239	3.048998	3.912409	3.253300	5.021277
Documents					
with B-Terms	9,202,628	14,329,391	1,842,409	5,293,385	62
with B- & C-Terms	4,484,726	7,586,298	819,922	2,578,483	43
Docs. with B- and C-Terms per					
Docs. with B-Terms	48.733%	52.942%	44.503%	48.711%	69.355%
Concepts per Document					
with B-Terms	0.000147	0.000188	0.000074	0.000301	2.274194
with B- & C-Terms	0.000303	0.000355	0.000167	0.000617	3.279070
Synonyms per Document					
with B-Terms	0.000332	0.000508	0.000288	0.000916	11.419355
with B- & C-Terms	0.000682	0.000960	0.000646	0.001880	16.465116

The number of documents containing terms extracted from ESSO corresponds to more than 90% of the total of processed documents meaning that the ESSO ontology includes terms that are the least specific for the analyzed corpus in comparison to the other ontologies. For the ontologies EpSO and EPILONT the coverage is slightly above half the size of the corpus or somewhat below the average, respectively. From a broad perspective, we could argue that the term set from EPILONT produces a rather narrow and possibly very specific set of documents from the 2021 BioASQ corpus while, by contrast, the term set from ESSO a very unspecific and a large document set.

The term set from EpSO has a slightly lower number of concepts in comparison to EPISEM, but generates a much larger retrieval of documents when considering either the *B-Terms* only or the co-occurrence of *B-* and *C-Terms*. From these results, we can conclude that EPISEM covers a more specific set of terms in comparison to EpSO. When comparing the document retrieval for the *B-Terms* against that for the *B-* and *C-Terms*, it turns out that sets of documents are proportional across the different source ontologies ranging from 44*%* for EPILONT to 69*%* for FENICS; however, the quota is higher if the source ontology covers a smaller amount of terms and concepts, i.e. when it could be considered to be more specific.

### Comparison of the source ontologies

String similarity on concept names and synonyms is used to get further insights from the term sets across the different ontologies. Each concept on the source ontologies holds a set of synonyms. Although not a common practice, a synonym for a concept can be reused by another concept within the same ontology. Reuse of synonyms across different ontologies is much more common and can help match concepts with each other. We therefore computed the synonym overlapping within and across the ontologies. A synonym is considered to overlap if it is used by two or more different concepts, showing a degree of similarity between them. Synonym-based similarity can be explicit or implicit. For instance, having a concept *b*_1_ with synonyms *s*_1_ and *s*_2_, a concept *b*_2_ with synonym *s*_1_ and a concept *b*_3_ with synonym *s*_2_ leads to (i) explicit similarities between *b*_1_ and *b*_2_ via *s*_1_ and between *b*_1_ and *b*_3_ via *s*_2_, but also to (ii) an implicit similarity between *b*_2_ and *b*_3_ because they are both, on its own, similar to *b*_1_.

**Fig. 3:**
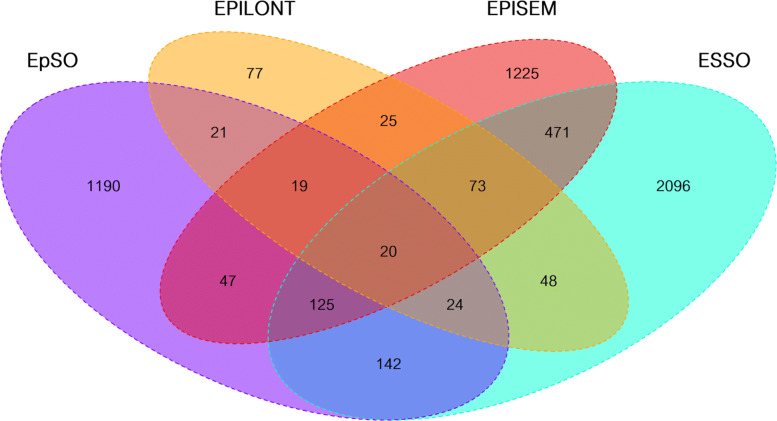
Quad Venn diagram for the concepts: Two concepts are considered to match if they share at least one synonym with each other. Set properties for FENICS are excluded as it is completely disjunctive to any of the other ontologies

The intersections between the source ontologies are visualized as a quad Venn diagram in Fig. 3. FENICS is omitted as it does not share any concept or synonym with any other source ontology. Furthermore, the resulting synonym-based mapping across the source ontologies is publicly available at NCBO Bioportal as Mapping of Epilepsy Ontologies (MEPO) [43].

Overall, the four sets of terms from the source ontologies cover 5,920 concepts with 5,603 unique concepts, out of which 4,588 concepts are only provided by a single source ontology, i.e. these concepts are not shared between any two ontologies. Concepts are considered to be redundant if they share one or more common synonym which applies to 317 concepts. This leaves 1,015 concepts that are shared between at least two source ontologies: 598 (22.197 *%*) out of the total number of concepts in the ontology of the concepts from ESSO are shared, 366 (23.004 *%*) from EPISEM, 167 (12.307 *%*) from EpSO, 60 (43.796 *%*) from EPILONT, and none for FENICS. EPISEM and ESSO share the biggest portion of concepts (689 concepts, i.e. 76.30 *%* of the shared concepts in ESSO and 88.33 *%* of those shared in EPISEM). These numbers show that FENICS is quite specific and therefore more useful for specialized retrieval, i.e. based on terms found only in this ontology, while EpSO and EPISEM could be used to narrow down results as they offer a low number of shared concepts. When comparing EPISEM against EpSO, the overlap is very small, only 39 concepts, i.e. they can be considered disjoint.

**Fig. 4:**
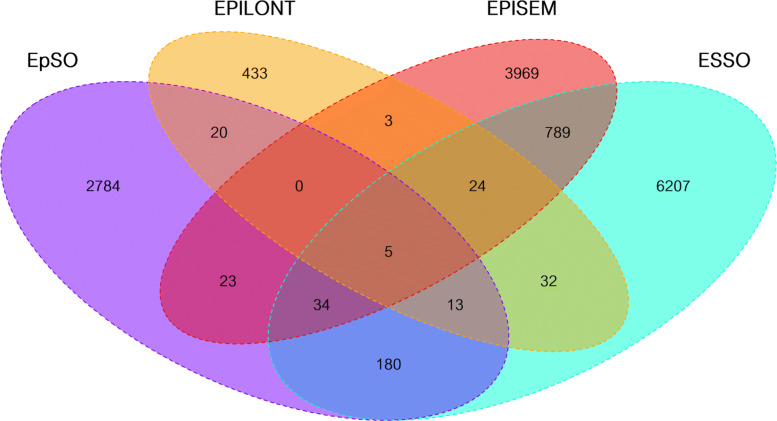
Quad Venn diagram for synonyms: All the synonyms from all concepts from each of the source ontologies are matched to each other. Again, FENICS is excluded as it is completely disjunctive

The shared synonyms from the source ontologies show a similar overall distribution when compared to the concepts one, see Fig. 4 (FENICS has been omitted as it is disjoint regarding all of the other ontologies). The ontologies provide 15,720 synonyms with 14,516 unique synonyms, out of which 13,393 synonyms are not shared between any two ontologies, i.e. about 92 *%*. From the remaining 1,123 shared synonyms, ESSO contains 1,077 shared synonyms (14.79 *%* out of the total synonyms in the ontology), EPISEM 878 (18.11 *%*), EpSO 275 (8.99 *%*), EPILONT 97 (18.30 *%*), and none from FENICS. ESSO has by far the largest amount of shared synonyms almost covering all of the shared synonyms, having the largest intersection of 852 synonyms with EPISEM followed by 232 shared synonyms with EpSO. In contrast, EPILONT has a low number of intersecting synonyms showing a disjunctive shape with regards to the other ontologies.

### Comparison of the document sets with B- and C-Terms

The 2021 BioASQ corpus was annotated with concepts from the source ontologies matching tokens of text with either preferred labels or synonyms. These annotations were used to compare the different subsets emerged from the of annotated documents. The proportion of shared concepts and synonyms from the source ontologies is also reflected in the document subsets tagged with terms from the respective ontologies, i.e. the set of documents containing *B-Terms*. Additionally, documents with co-occurring drug names are also taken into consideration for their representation of document subsets in the corpus, i.e. documents containing B- and *C-Terms*.

**Fig. 5:**
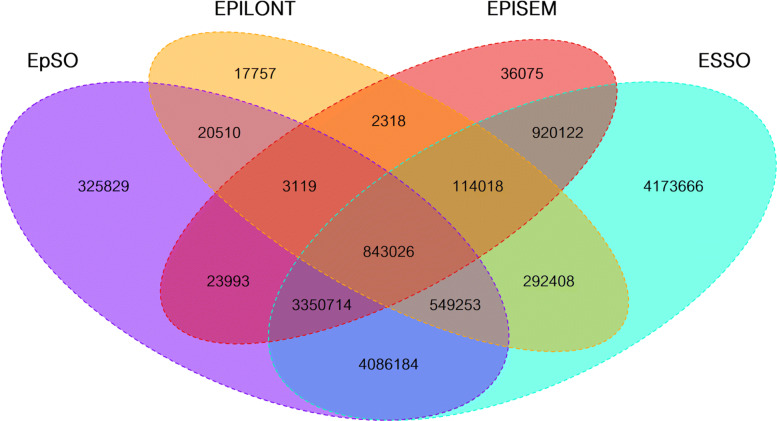
Quad Venn diagram for documents with *B-Terms*: The documents containing annotations from a source ontology are matched against each other. FENICS is excluded as terms from it are recognized only in two documents

From the 15,501,443 documents in the 2021 BioASQ corpus, 14,759,054 (95.21 *%*) documents were annotated with *B-Terms* from which 10,205,663 (69.15 *%*) are shared by at least two ontologies. There are only two documents exclusively annotated by FENICS omitted hereafter for being too specific. ESSO has terms occurring in 5,982,059 (58.90 *%*) documents with terms from other ontologies whereas EPISEM has co-occurring terms in 5,221,235 (99.31 *%*) documents with other ontologies, EpSO 8.550.970 (96.33 *%*) and EPILONT 1,806,895 (99.02 *%*). This shows that ESSO adds a very large amount of documents to the set of documents regarding the *B-Terms* leading to low specificity as it covers almost the entire corpus of documents. Moreover, EpSO and EPISEM also share a very large amount of several million documents in the corpus that appear to be very general and unrelated to the domain of epilepsy which is probably caused by highly common terms that are not specific to epilepsy. From the source ontologies, it is EPILONT the one adding more specific documents to the set of those that can be used for *B-Terms*. In Fig. 5, documents containing *B-Terms* are visualized as quad Venn diagram.

**Fig. 6:**
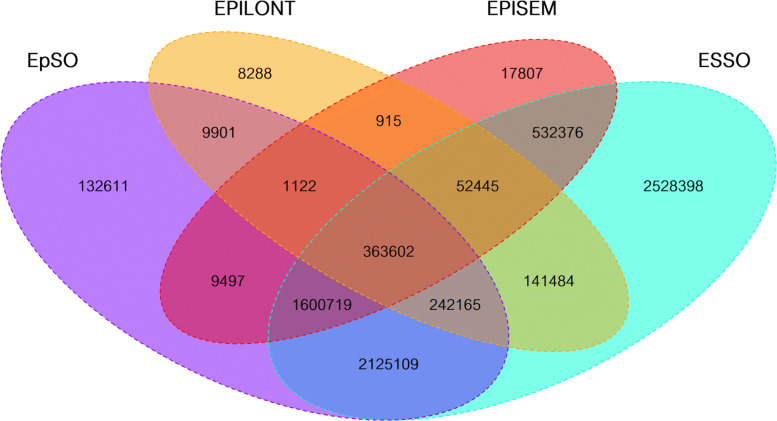
Quad Venn diagram for documents with *B-* and *C-Terms*: The documents containing at least one term from an ontology with at least one co-occurring drug name in the same document are matched against each other. FENICS is excluded due to its almost complete disjunctiveness

The document set containing *B-Terms* co-occurring with *C-Terms* from the Drug Name vocabulary shows a similar proportion of shared documents regarding *B-Terms* only. Most of the source ontologies contribute to the identification of about 97 *%* of documents containing drug names, i.e. *C-Terms*, except for ESSO which contributes only to 66.67 *%* of these documents. This large fraction of documents corresponding to ESSO annotations not shared with any of the other source ontologies suggests that ESSO is not specific enough for document retrieval regarding epilepsy. Similarly, EpSO and EPISEM appear to add quite general documents to the set of annotated documents with several million of documents there. EPILONT is the only ontology whose annotations suggest a good specificity for document retrieval aligned to epilepsy, with only 811,634 documents where drug names, i.e. *C-Terms*, co-occur with *B-Terms* in the 2021 BioASQ corpus. Nevertheless, most of these documents with EPILONT annotations also exhibit annotations from other ontologies. In Fig. 6, the quad Venn diagram shows documents containing *B-Terms* co-occurring with *C-Terms*.

### Prioritization of drugs

The extracted drugs are sorted according to the document frequency and their co-occurrence with *B-Terms*; they are stored into ranked lists of drug names. One ranked list is produced per ontology containing 8,010 drugs for EpSO, 8,620 for ESSO, 6,318 for EPILONT, 7,641 for EPISEM and 74 for FENICS. Repurposing candidate drugs for the neurological domain requires further scrutiny than drugs for other pathological indications. One common constraint for neurological drugs is to be able to pass the blood-brain barrier. Therefore, the ranked lists are filtered by drug names classified as relevant to the Nervous System according to the Anatomical Therapeutic Chemical (ATC) Classification System’s [44] class *N*. Table 2 shows the top 25 drug names per ranked list, with FENICS showing only four drug names after the filtering process. The total length of the ranked lists of drug names after the filtering process selecting only neurological drugs is 465 for EpSO, 471 for ESSO, 431 for EPILONT, 465 for EPISEM and 6 for FENICS. The lists of drug names produced per ontology are composed approximately by the same drugs before as well as after the filtering process (except for FENICS). The major difference between the lists is not the varying number of drugs but the ranking of those drugs within these ontology-derived lists.

**Table 2:** The top-25 most frequently co-occurring neurological drugs with terms wrt the epilepsy ontologies. The list of FENICS has only five entries as there are no more co-occurring neurological drug names in FENICS

Rank	EpSO	ESSO	EPILONT	EPISEM	FENICS
1	Ketamine	Ketamine	Ketamine	Morphine	Phenytoin
2	Morphine	Morphine	Valproic acid	Ketamine	Caffeine
3	Nicotine	Tryptophan	Carbamazepine	Nicotine	Eslicarbazepine
4	Dextroamphetamine	Diethyl ether	Levodopa	Levodopa	Oxcarbazepine
5	Tryptophan	Nicotine	Morphine	Lidocaine	Carbamazepine
6	Diazepam	Caffeine	Nicotine	Naloxone	Disulfiram
7	Haloperidol	Naloxone	Phenytoin	Fentanyl	
8	Amphetamine	Lidocaine	Diazepam	Acetaminophen	
9	Phenobarbital	Acetaminophen	Tryptophan	Tryptophan	
10	Levodopa	Melatonin	Propofol	Capsaicin	
11	Diethyl ether	Dextroamphetamine	Phenobarbital	Levobupivacaine	
12	Valproic acid	Propofol	Caffeine	Dextroamphetamine	
13	Naloxone	Levodopa	Naloxone	Diazepam	
14	Carbamazepine	Phenobarbital	Lidocaine	Propofol	
15	Lidocaine	Diazepam	Haloperidol	Bupivacaine	
16	Melatonin	Chloroform	Esketamine	Caffeine	
17	Caffeine	Fentanyl	Dextroamphetamine	Melatonin	
18	Propofol	Haloperidol	Pentobarbital	Haloperidol	
19	Acetaminophen	Amphetamine	Diethyl ether	Esketamine	
20	Fentanyl	Pentobarbital	Lamotrigine	Valproic acid	
21	Fluoxetine	Esketamine	Midazolam	Amphetamine	
22	Esketamine	Valproic acid	Levetiracetam	Diethyl ether	
23	Phenytoin	Capsaicin	Melatonin	Phenobarbital	
24	Pentobarbital	Levobupivacaine	Amphetamine	Pentobarbital	
25	Clozapine	Isoflurane	Dalfampridine	Midazolam	

The results show that each of the different ontologies prioritize drugs related to the treatment of epilepsy (e.g. Ketamine), the treatment of the nerve system (e.g. Fentanyl) or known to generate epileptic reactions (e.g. Lidocaine). Notingly, Capsaicin appears in the ranking for EPISEM as a pharmaceutical compound with neuroprotective properties in the context of seizure prevention in rodent models [45, 46]. Furthermore, the five ranked list of drug names are combined into a final ranked list using an alignment algorithm to determine the prioritization of the drugs based on our ABC discovery approach. The alignment algorithm minimizes the distances used for the final ranking.

Table 3 shows the final combined ranked list of drug names, including columns for priority, the intersections with co-occurrences in the source ontologies, the type of relation with epilepsy, the ATC class, and the match with a reference set. The first column, *Priority*, provides an informative measure on how many intersections match to the reference lists and the ATC classes *Anti*−*Epileptics*(*N*03), having the optimal *Score*=5 when there is an intersection with all four reference lists and the ATC class N03.

**Table 3:**
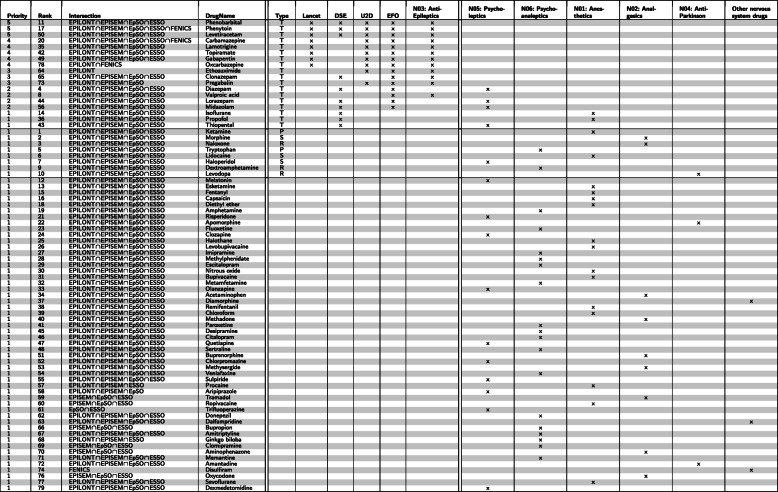
The final ranked list of drug names co-occurring with terms from the respective Epilepsy Ontologies showing columns for ATC classes as well as matches with reference lists: DRUGSE from the journal Drug [38], EFO [39], U2D [40, 53], Lancet from the journal Lancet [37]. The score in the first column is based on the number of reference lists recommending the drug for the treatment of epilepsy. The column “Rank” provides the original rank from the aggregation of the combined ranked list according to the TopKLists calculations. The column “Type” refers to wether the drug can be categorized to either “T: Certainly used for treating epilepsy”, “P: Potentially be used for treating epilepsy”, “R: Having some kind of relation to epilepsy” and “S: Causing epilepsy as side effect.” The Type is only provided for scores above 1 and for drugs ranked within the top 10 of the final combined ranked list

Table 3 compares the ranking of well-established drugs according to known reference sets (uppert part), against drugs with a high ranking according the presented approaches (middle part) and the remaining known drugs. This comparison allows the assessment of well-established drugs (known from different sources) with drugs from the literature, where the thereapeutic benefits are more questionable. To judge the findings from the table, we chose Phenobarbital, which has a priority score of 5, and is a well-established drug for epiplepsy and thus well known to the reference resources. For the substance Dextroamphetamine we see a ranking of 9 (in the top 10) but it does not appear in any of the reference sets. Last, Melatonin has the highest rank at position 12 with a priority of 1. In summary, the upper part of Table 3 is sorted by priority while the middle part by rank. Since the suggested approach does not differenciate between drugs used for treatment and drugs having epilepsy as side effect, thus being active on the neural system, we can conclude that the lower part comprises alternative candidates for drug repurposing.

The reference lists come from the journal Lancet [37] (reference data set called Lancet), the journal Drugs [38] (reference data set called DRUGSE), the Epilepsy Foundation website [39] (reference data set called EFO), and an updated list of epilepsy drugs [40] (reference data set called U2D). Enriched plots for the ranked lists comparing them to the reference lists are used for the evaluation. Furthermore, an enrichment analysis is used to compare the lists ranking regarding to the union of the four reference sets Lancet, DRUGSE, EFO, and U2D.

**Fig. 7:**
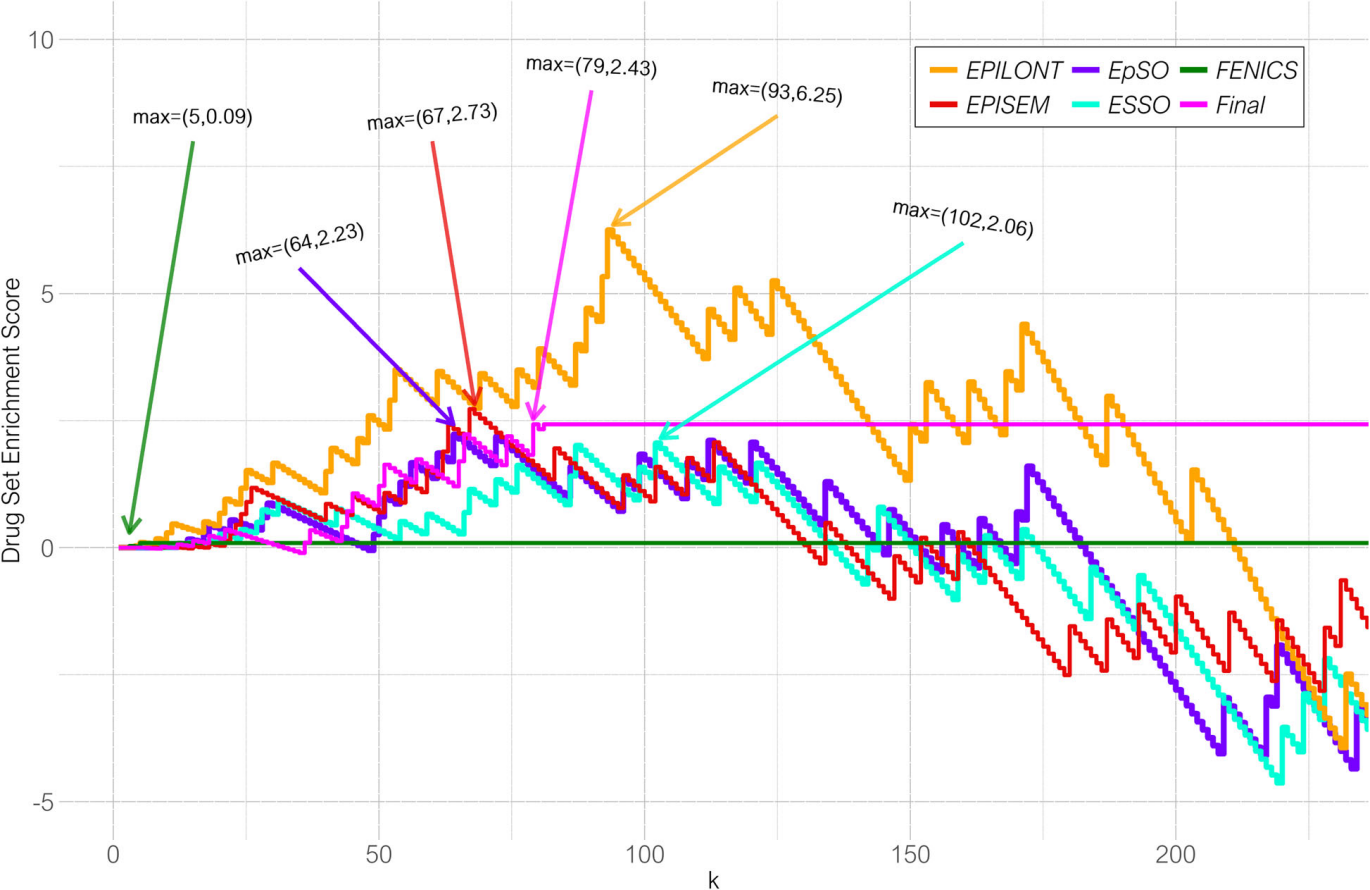
For the comparison of different ranked lists, enrichment scoring provides an impression of how much a ranked list of entities intersects with a reference list by the step-wise increment. The Drug Set Enrichment Scoring function is a specialized enrichment scoring function that calculates bonuses and penalties with an adaptive step size adjusted for short lists. Here, the reference list is composed of drugs that are used in the clinical practice for epilepsy. The Drug Set Enrichment Scores for the five ranked lists of drug names produced by each of the epilepsy ontologies visualized the enrichment in comparison to the reference list. Additionally, the scores for the final combined ranked list are visualized in magenta color indicated as "Final". Maximum values are marked with arrows for x and y values

The Drug Set Enrichment Analysis provides a scoring for each of the ranked lists for the respective epilepsy ontologies as well as for the final combined ranked list by adding either a bonus for a match or a penalty for a miss with regards to the reference list. In Fig. 7, the drug set enrichment score is plotted for each of the five ranked list and for the final combined ranked list including a marker for the maximum drug set enrichment score with its position *k*. The ranked list of drug names for EPILONT shows the highest enrichment score with a maximum at position *k*=93 doubling out the maximum score from any of the other ranked lists, including the final combined ranked list. This shows that, although the dictionary of terms from the EPILONT with 137 concepts and 530 synonyms is rather small in comparison to the ones for EpSO, ESSO, and EPISEM, the co-occurring drug names show a high specificity for relevant drug names for epilepsy.

## Discussion

Other text mining approaches on literature-based discovery for drug repurposing, e.g. [47–49], also provide rankings for the extracted drug disease associations evaluated by comparing them to factual databases, e.g. the Comparative Toxicogenomics Database (CTD) [50, 51], or to expert judgment on the significance of the involved biological pathways. These types of evaluation follow the assumption that biological interactions, e.g. biological pathways, support the identification of drug disease associations. As the mechanism of action is often unknown, especially for neurological drugs, it is also not known which neurological drugs potentially target the common underlying malfunctioning neurological processes shared between different types of neurodegenerative diseases.

The approach described in this work differs from existing approaches as it identifies implicit relationships and then aggregates several ranked lists of identified drug names. Furthermore, the ranked lists of drug names are enriched by incorporating the novel drug set scoring method. The results presented in this paper provide an overview of the relevant drug names for the entire domain of epilepsy and adjunct neurological disorders.

### Drug associations in the context of epilepsy

The drug associations in the context of epilepsy are further explored by the manual inspection of the information available about the mode of actions on the DrugBank database as well as from literature search where each evidence is cited accordingly. The ranked and extracted drug names can be categorized with regards to their relation to epilepsy. The assigned categories are: (i) drugs being certainly used for epilepsy, (ii) potentially used for epilepsy, (iii) having some type of relation to epilepsy or (iv) causing seizures as side effect. The properties of the drugs can be derived from their classification according to ATC, evidence in literature or descriptions of side effects and mechanism of action on databases such as DrugBank [52]. In the following, the top 10 drugs from the combined ranked list are discussed together with their association to epilepsy.

#### Drugs certainly used for treating epilepsy

Phenobarbital can certainly be used for the treatment of epilepsy as it has been approved as an anti-epileptic drug while Diazepam is recommended in the reference sets DSE and EFO. Phenobartibal is used for seizure control for all types of seizures except absent seizures. Diazepam is a psycholeptic drug used for the treatment of seizures due to its anticonvulsant effects.

#### Drugs potentially used for treating epilepsy

Drugs with a potential use for epilepsy are Tryptophan and Ketamine. Tryptophan potentiates serotonin release in brain activity which can also improve seizure control in refractory epilepsy [54]. Ketamine is a common anesthetic which was later approved for refractory depression [34]; it has also shown efficacy for the management of refractory epilepsy [35].

#### Drugs having some relation to epilepsy

The administration of drugs affecting the activity of neurological receptors such as dopamine receptors has a potential effect on the onset of epilepsy. Children with attention deficit disorder having epilepsy as co-morbodity are speculated to be safe for being treated with Dextroamphetamine; however, controlled studies about the effect of Dextroamphetamine in patients with epilepsy are still missing [55]. Levodopa causes an increase of dopamine release useful to reduce the effects of Parkinson’s Disease but the clinical effects of Levodopa on epilepsy patients is barely studied [56]. As opiod-derivatives such as Diazepam are used for seizure control, the administration of Naloxone might be used for epilepsy patients because it is an opioid antagonist medication that blocks or reverses the effects of opioid drugs [57].

#### Drugs causing seizures as adverse effect

Some medications cause seizures as adverse effects. Lidocaine can cause seizures in patients having a history of epilepsy [58]. Antipsychotic drugs such as Haloperidol are associated to lowering the threshold of seizures and potentially induce seizures [59]. Opiod therapy with Morphine might cause seizures but the effect is still poorly understood [60].

### Limitations

Although drugs frequently have an off-label use, the major limitation of drug repurposing approaches is that the actual approval of a drug for a new disease indication still has to undergo clinical testing. Nevertheless, automated workflows enable domain experts to quickly evaluate validation approaches. The ranking and aggregation of drug names with the aggregation of a top-k ranked list as well as the drug set enrichment analysis scoring function allow for a graphic interpretation of the neurological drug space in the context of epilepsy. This can easily be transferred to other disease indications by incorporating other domain-specific ontologies for the extraction of relevant chemical compounds for drug repurposing such as for the viral domain.

Our tailored implementation of the Open Discovery Process is narrowed down to the domain of epilepsy allowing for manual inspection of the mode of action (such as a drug used on treatments for epilepsy or causing seizures) of the top ranked drug names in high-quality databases such as DrugBank. This manual inspection would still be possible when applying our method to different diseases as DrugBank, for instance, also includes drugs for other diseases. In case of a generalization to the a broader coverage of neurological diseases, the type of relation will still be dependent on the respective disease and potentially require the automated detection of relationship types by using methods such as the one proposed by [61]. Nevertheless, the incorporation of further expert knowledge from curated databases provides an opportunity to improve the automated detection of relationship types in the free text of biomedical publications which could include some mismatches (as any other prediction method).

However, neurological drugs which do not have many mentions in the literature but contrastingly have a potential relevance for the domain of epilepsy will hardly be identified with our approach. The major measurement for the implication of relevance in our approach is based on frequent occurrences of terms in documents. As clinical practice as well as related scientific publications are purely dependent on the opinion of medical practitioners, their current view on the pharmacology of epilepsy will be taken into account. This will potentially neglect pharmaceutical compounds that were not in the focus of current clinical practice or by related scientific publications.

Furthermore, the filtering step excluding any non-neurological drugs from further investigation might drop-out drugs that are not yet identified for their efficacy in the neurological domain. For example, certain drugs which are improved for the treatment of organ diseases could have an unknown effect on the brain. Future work could include the investigations of neurological side effects of non-neurological drugs in order to identify such a potential efficacy for neuropathological disorders and diseases.

### Outlook

With the increased risk of epidemiological outbreaks, novel approaches for drug repurposing incorporating statistical evidence derived from large-scale literature analysis are urgently needed. Drug repurposing is particularly valuable for rare or new diseases, e.g. COVID-19 in 2019, as there is not enough clinical data that can be reliably used. New techniques, e.g. RNA-based, to produce vaccines has also been favored in recent years as their development process can go faster than traditional methods. The use of existing biomedical ontologies for text mining on biomedical literature and its combination with knowledge contained in biological databases becomes an in-silico asset to fight diseases via drug repurposing. As future work, the incorporation of more biomedical ontologies into the Open Discovery Process will allow for a wider range of applications. Especially, the incorporation of large ontologies such as the International Classification for Drugs and Drug Dosages (ICD) or SNOMED-CT will be of greater interest. Also, the idea behind the presented work is the use of complete ontologies with a domain focus. This is new and gives new opportunities in the future. A broad disease ontology, like SNOMED-CT will be explored in future experiments, but this approach used only ontologies that are definitely focused to epilepsy only.

## Conclusions

Text Mining can contribute to the process of drug repurposing by providing empirical evidence about the similarity of entities related to drugs and diseases. The Open Discovery Process is a systematic approach to find implicit relationships between previously unrelated concepts. In this project, it has been used with textual evidence of *B-Terms* from epilepsy-specific domain ontologies co-occurring with drug names. The data analysis on the drug set enrichment scoring function and used to compare the ranked result lists of drug names is a novel approach to incorporate ontologies for the extraction of relevant drug names for drug repurposing. The extracted ranked list of drug names from the literature could reduce the amount of time and money spent for the pre-clinical stratification of new applications of neurological drugs for epilepsy.

The retrieval of biomedical documents shows a high diversity where the use of domain-specific ontologies provide the advantage of having a high coverage with regards to epilepsy as well as drugs. The ranking of drug names in these documents provides a more tuned retrieval towards other types of drugs than the general occurrence of common drug names. This ranking of drug names specific to the domain of epilepsy can provide benefits to patients by giving an overview of potential drugs for their disease indications. Furthermore, the ranking is also relevant for researchers in order to identify drugs for epilepsy as well as epilepsy-related drug names.

Future work will incorporate recent advancements in natural language processing and further sets of drug names beyond those identified as neurological drug names according to ATC. This will overcome the limitations related to the filtering by neurological drug names only. Furthermore, the construction of a hybrid named entity recognition system (hNER) making use of dictionaries and pre-trained language models will potentially provide a substantial boost in performance. Additionally, more generalized ontologies would also cover a broader spectrum of potentially novel relations between existing drug names and diseases and disorders. Further evaluation against similar approaches and corpora will also be included in our next iteration.

## Methodology

The main goal of this work is creating a list of candidate drugs for epilepsy. This is achieved by following the model of the Open Discovery Process, i.e., in this case, connecting the disease epilepsy as set *A* through *B-Terms* from the epilepsy ontologies with a resulting set *C* of drug names. The top-k repurposing candidate drugs for epilepsy are calculated based on the co-occurrence frequency regarding terms from the epilepsy-specific domain ontologies, i.e. EpSO, ESSO, EPILONT, EPISEM, and FENICS, identified in the 2021 BioASQ corpus corresponding to 15,501,443 articles from Medline [62].

**Fig. 8:**
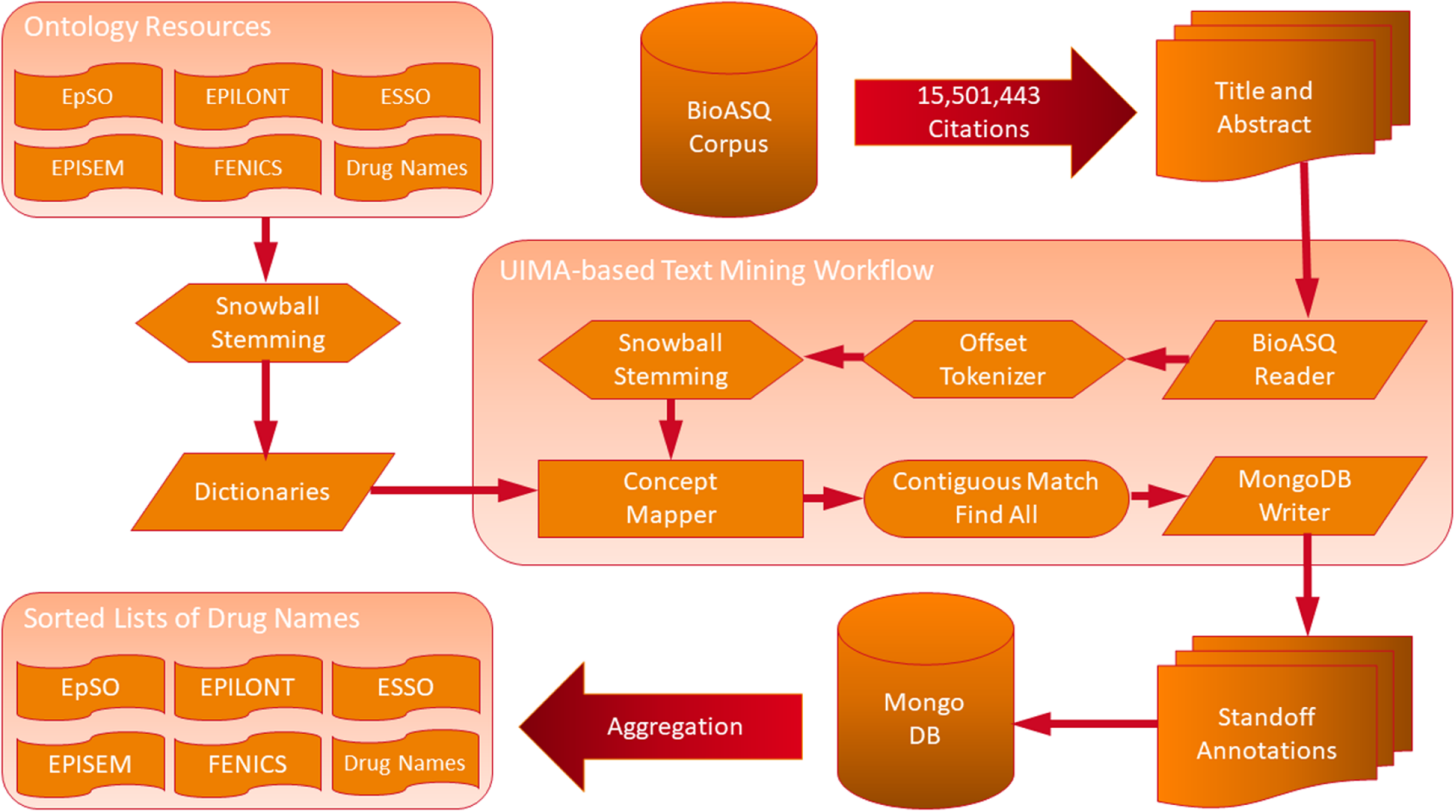
The UIMA-based text mining workflow reads all documents from the 2021 BioASQ corpus in order to tokenize, stem, and annotate them with terms from the ontologies EpSO, ESSO, EPILONT, EPISEM, FENICS, an DrugBank. The resulting annotations are stored in MongoDB and later used for aggregating the frequency of co-occurring drug names for each of the ontologies

The initial step is creating dictionaries for each of the ontologies and drug name sources; these dictionaries are used as part of a NER pipeline to extract drug names co-occurring with terms from the epilepsy ontologies. The annotations obtained from the NER pipeline are stored and used to create drug candidate lists per each ontology. These lists are combined into a ranked list containing the best drug candidates according to the Open Discovery Process. The final step consists in scoring the drug candidate regarding a reference set of drug names. The overview is depicted in Fig. 8.

In the following, the different components of the methodology are explained in more detail including the creation of the dictionaries, the text mining workflow for recognizing terms in publications, and the data analysis using R.

### Approach

The dictionaries for each of the ontologies (EpSO, ESSO, EPILONT, EPISEM, and FENICS) as well as drug names are created by extracting names, labels, and synonyms from the respective source files; additional synonyms are created with the snowball stemming algorithm [63]. The ontologies were downloaded in OWL format from BioPortal [8], while the DrugBank vocabulary in XML format was obtained from the DrugBank Open Data data set [52]. All the generated dictionaries are available online [64].

A UIMA [65]-based text mining workflow is used for NER on documents from the 2021 BioASQ corpus. This workflow has also been used to annotate life science entities with the UIMA ConceptMapper [66] in the corpus behind the search engine LIVIVO [67, 68], and evaluated for their use on literature information retrieval [69]. The workflow was extended for its application on the BioASQ challenge using a pre-trained language model. The goal there was re-ranking MeSH terms found in Medline citations according to their term similarity, resulting in a boost of performance [70]. The NER process used in the present work, including reading the documents and writing the annotations, took a total runtime of 7.73 hours on a laptop.

The annotations corresponding to the different dictionaries are written into a MongoDB [71] collection with a size of 43.3 Gigabytes for later analyses. Every time that a drug name co-occurs with at least one ontology term in the same document is counted as a hit and recorded in MongoDB. This results in five MongoDB drug name aggregations, one per source ontology. In this way it is possible to link drug names, i.e. *C-Terms* to epilepsy via epilepsy-related *B-Terms*. The annotated corpus with its aggregations is available online as BioASQ Sub-Corpus for the Pharmacology of Epilepsy (BioPepsy) [72].

The five MongoDB aggregations are processed and analyzed with the R-package *epos* [73, 74]. Lists are created out of the aggregations and drug names are sorted based on their document frequency.

These drug name sorted lists are filtered according to ATC so only neurological drugs are retained. The R-package *TopKLists* is used [75] to combined the sorted and filtered lists into one final ranked list. TopKLists uses the Cross Entropy Monte Carlo algorithm to rank the combined element and determine the optimal resulting length *k*. Table 3 presents the final ranked list including the final score, the rank within the combined list, the ontologies where co-occurring terms were identified, the drug name and additional information regarding reference lists of epilepsy-related drug names and ATC classes. The score on the first column is calculated using the Drug Set Enrichment Analysis (DSEA), see the paragraph below. A summary of the DSEA score for the final ranked list as well as the individual ontologies is visualized in Fig. 7.

The DSEA was particularly developed for the work presented in this manuscript. It calculates and assigns a score to elements of a drug ranked list based on the comparison against a reference set of drug names. Matches to the reference set are favored with a bonus while mismatches are penalized. The DSEA score is similar to the gene set enrichment analysis (GSEA) score used for gene expression data sets [76, 77]. In comparison to the GSEA score, the DSEA scoring function is more relaxed with regards to a shorter length of the sets. In particular, it uses an adaptive variable *τ* as controlling parameter for adjusting the penalty and bonus with an increasing length of the list, as shown in Eq. 1 and corresponding Algorithm 1.

1 $$  \begin{aligned} \text{For}\ \, D = \{d_{1}, \dots, d_{N}\};\ R &= \{r_{1}, \dots, r_{S}\};\ x_{0}=0\\ DSEA(D,R) \mapsto \Psi (x) &= \sum_{i=1}^{N} (x_{i-1} + \vartheta) \\ \text{with}\ \vartheta &= \begin{array}{ll} \ln \left(\frac{S-\tau}{S+\tau}\right), & \text{if}\ d_{i} \in R\\ \ln \left(\frac{N-\tau}{N+\tau}\right), & \text{otherwise} \end{array} \\ \text{with}\ \tau &= \sum_{j=1}^{j=i}|d_{j} \in R| \end{aligned}  $$

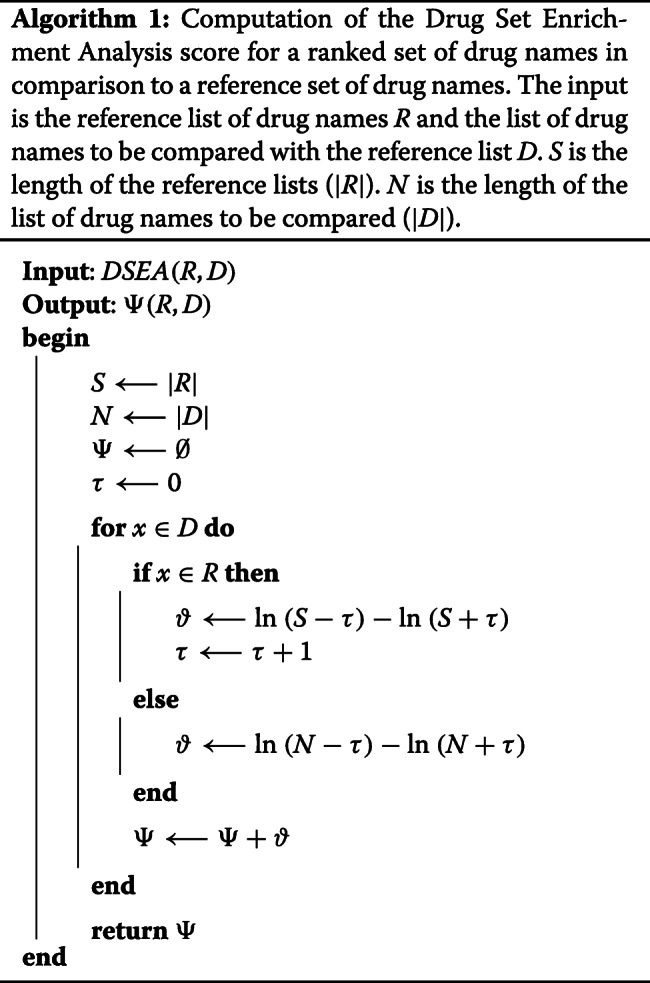


### Implementation and availability

The implementations in JAVA are published on GitHub as part of the Project SNOKE, a frozen version has been archived and is publicly available [78]. The implementations creating the dictionaries from the ontologies are available at the module snoke.ontology while the components for the UIMA-framework at the module snoke.uima. Documentation for all the modules is available as Javadoc.

The source code for the statistical analysis together with the analysed data sets are published on the Comprehensive R Archive Network (CRAN) as the R-package EPOS, a frozen version has been archived and is publicly available [73, 74]. The published data sets comprise the reference sets of drug names as well as the ranked list of drug names co-occurring with terms from the source ontologies EpSO, ESSO, EPILONT, EPISEM, and FENICS, and archived version is available [79]. The source code also contains the implementation of the algorithm for the DSEA scoring function. Additionally, the package contains unit tests as well as documentation of the source code.

## Data Availability

The *BioASQ Sub-Corpus for the Pharmacology of Epilepsy (BioPepsy)* is published under [72]. The Java software *Towards a Semantic NoSQL (Not only SQL) Knowledge Environment (SNOKE)* is published under [78]. The R package *epos: Epilepsy Ontologies’ Similarities* is published on the Comprehensive R-Archiving Network (CRAN) under [73] and as *R-package for the Analysis and Visualization of Epilepsy Ontologies’ Similarities According to Co-Occurring Drug Names in the 2021 BioASQ corpus* under [74]. The dictionaries for the UIMA ConceptMapper are published as *UIMA ConceptMapper Dictionaries for the Annotation of the 2021 BioASQ Corpus with Drug Names and Terms from Epilepsy Ontologies* under [79]. The mapping between the ontologies is published as *Mapping of Epilepsy Ontologies (MEPO)* under [43].

## Abbreviations

ArrayExpress: the Archive of Functional Genomics Data
ATC: the Anatomical Therapeutic Chemical Classification System
BioASQ: biomedical semantic indexing and question answering
BioPepsy: BioASQ Sub-Corpus for the Pharmacology of Epilepsy
CRAN: the Comprehensive R Archive Network
CTD: Comparative Toxicogenomics Database
DisGeNET: the database of gene-disease associations
DrugBank: the database DrugBank
DRUGSE: reference data set from the journal Drugs
DSEA: Drug Set Enrichment Analysis
EFO: reference data set from the Epilepsy Foundation
EPILONT: the Epilepsy Ontology
EPISEM: the Epilepsy Semiology
epos: the Epilepsy Ontologies’Similarities
EpSO: the Epilepsy and Seizure Ontology
ESSO: the Epilepsy Syndrome Seizure Ontology
FDA: US Food and Drug Administration
FENICS: the ontology Functional Epilepsy Nomenclature for Ion Channels
GEO: the database Gene Expression Omnibus
GSEA: Gene Set Enrichment Analysis
KEGG: Kyoto Encyclopedia of Genes and Genomes
Lancet: reference data set from the journal Lancet
Medline: the database Medline
MeSH: Medical Subject Headings
MongoDB: huMONGOus Database
N01: therapeutic subgroup Anesthetics from ATC
N02: therapeutic subgroup Analgesics from ATC
N03: therapeutic subgroup Antiepileptics from ATC
N04: therapeutic subgroup Anti-parkinson drugs from ATC
N05: therapeutic subgroup Psycholeptics from ATC
N06: therapeutic subgroup Psychoanaleptics from ATC
N07: therapeutic subgroup Other nervous system drugs from ATC
NCBO: National Center of Biomedical Ontologies
NER: Named Entity Recognition
NLM: National Library of Medicine
OMIM: the database on Online Mendelian Inheritance in Man
PubChem: the database PubChem
PubMed: the database PubMed
SARS-CoV-2: severe acute respiratory syndrome coronavirus 2
SIDER: the database Side Effect Resource
SNOKE: Semantic NoSQL Knowledge Environment
U2D: reference data set from UpToDate.com
UIMA: Unstructured Information Management Architecture
